# A new, alternative risk score for sarcopenia in Chinese patients with type 2 diabetes mellitus

**DOI:** 10.1186/s40001-023-01127-1

**Published:** 2023-05-10

**Authors:** Qinghua He, Xiuzhi Wang, Caizhe Yang, Xiaoming Zhuang, Yanfen Yue, Hongjiang Jing, Jing Hu, Mingxiao Sun, Lixin Guo

**Affiliations:** 1 Department of Endocrinology, Beijing Hospital, National Center of Gerontology, Institute of Geriatric Medicine, Chinese Academy of Medical Sciences, Beijing, 100730 China; 2 Department of Endocrinology, Pinggu Hospital, Beijing Traditional Chinese Medicine Hospital, Beijing, 10120 China; 3 Department of Endocrinology, Air Force Characteristic Medical Center, Beijing, 100142 China; 4 Department of Endocrinology, Fuxing Hospital Affiliated to Capital Medical University, Beijing, 100038 China; 5 Department of Nutrition, Pinggu Hospital, Beijing Traditional Chinese Medicine Hospital, Beijing, 10120 China; 6 Department of Nutrition, Air Force Characteristic Medical Center, Beijing, 100142 China; 7 Department of Nutrition, Fuxing Hospital Affiliated to Capital Medical University, Beijing, 100038 China; 8 Department of Endocrinology, Beijing Yide Hospital, Beijing, 100195 China

**Keywords:** Sarcopenia, Type 2 diabetes, Risk score

## Abstract

**Objective:**

To develop a new, alternative sarcopenia risk score to screen for sarcopenia in type 2 diabetes patients in China and to demonstrate its validity.

**Research design and methods:**

The data for this study came from a multicenter, cross-sectional study that had been designed to estimate the prevalence of sarcopenia among adults with type 2 diabetes and had been conducted in several hospitals in Beijing, China. A total of 1125 participants were randomly divided into two groups: an exploratory population and a validation population. A multivariable logistic regression model using the backward stepwise likelihood ratio method to estimate the probability of sarcopenia was fitted with candidate variables in the exploratory population. A new, alternative sarcopenia risk score was developed based on the multivariable model. The internal and external validations were performed in the exploratory and validation populations. The study was registered at Chinese Clinical Trial Registry (ChiCTR-EOC-15006901).

**Results:**

The new, alternative sarcopenia risk score included five variables: age, gender, BMI, total energy intake per day, and the proportion of calories supplied by protein. The score ranged from − 2 to 19. The area under the receiver operating characteristic (ROC) curve of the risk score for the prediction of sarcopenia in type 2 diabetes patients was 0.806 (95% CI 0.741–0.872) and 0.836 (95% CI 0.781–0.892) in the exploratory and validation populations, respectively. At the optimal cutoff value of 12, the sensitivity and specificity of the score for the prediction of sarcopenia were 70.9% and 81.0% in the exploratory population and 53.7% and 88.8% in the validation population, respectively. The Hosmer–Lemeshow goodness-of-fit test showed a good calibration with the risk score in external validation (*χ*^2^ = 4.459, *P* = 0.813).

**Conclusions:**

The new, alternative sarcopenia risk score appears to be an effective screening tool for identification of sarcopenia in Chinese patients with type 2 diabetes in clinical practice.

*Clinical trial registration* Chinese Clinical Trial Registry, ChiCTR-EOC-15006901.

## Introduction

Sarcopenia has been described as an age-related decline in muscle function (defined by muscle strength or physical performance) and in skeletal muscle mass that may result in increased disability and mortality [1]. Currently, sarcopenia has been accepted as a new geriatric syndrome and has become an important phase in the transition to geriatric frailty syndrome [2]. Sarcopenia is treatable and may be preventable [1, 3]. It is realized that interventions might be more effective early than late in the course of developing physical disability or functional dependence [4]. The early stage of sarcopenia before physical disability or functional dependence might therefore represent a valuable opportunity to conduct interventions to decelerate the progress of sarcopenia and prevent physical disability [5, 6]. However, sarcopenia has been overlooked and undertreated in mainstream practice, apparently due to the complexity of determining what parameters to measure, how to measure them, what cutoff points best guide diagnosis and treatment, and how to best evaluate therapeutic effects. Sarcopenia patients are generally unaware of their sarcopenic state until the gradual reduction in muscle mass and function becomes very serious and leads to physical and functional disability. Therefore, routine medical screening to detect sarcopenia before the occurrence of physical disability could improve the chance of intervention.

According to current international consensus from the European Working Group on Sarcopenia in Older People (EWGSOP) [3, 7], the Asian Working Group for Sarcopenia (AWGS) [8], the International Working Group on Sarcopenia (IWGS) [9], the recommended criteria for the diagnosis of sarcopenia require the detection of low muscle strength or low physical performance and low muscle mass. Muscle strength is commonly assessed with handgrip strength, and physical performance is assessed with the Short Physical Performance Battery or usual gait speed; muscle mass is estimated by bioimpedance analysis (BIA) or dual-energy X-ray absorptiometry (DXA). The diagnosis of sarcopenia is device-dependent and time-consuming, and sometimes the feasibility is limited by the need for special equipment and training in many clinical settings. Therefore, a brief and alternative screening tool for sarcopenia that does not depend on many instruments is required. The SARC-F [10] is a 5-item questionnaire used to screen for sarcopenia risk and is self-reported by patients. Responses are based on the patient’s perception of limitations in strength, walking ability, rising from a chair, stair climbing and experiences with falls. The SARC-F is suitable for large-scale population screening in epidemiological studies, however, has been criticized for its low to moderate sensitivity which results in false negative results for sarcopenia screening, makes it is not a very ideal screening test for sarcopenia. The Mini Sarcopenia Risk Assessment (MSRA) [11] is also a simple questionnaire to rapidly screen high-risk sarcopenia patients, particularly in community-dwelling older adults, need to be tested for their performance and usability with future studies in different populations and settings. Type 2 diabetes mellitus increases the risk for impaired mobility and strength and is thought to be an important predictive factor of sarcopenia [12, 13]. Type 2 diabetes is associated with lower skeletal muscle strength and quality as well as the excessive loss of skeletal muscle mass and increases the risk of developing sarcopenia [14]. Studies have shown that the prevalence of sarcopenia in Korean patients with diabetes was higher than that in non-diabetes patients (15.7 and 6.9%, respectively). In China, the prevalence of adult diabetes has reached 10.4% [15], indicating a very large number of diabetes patients in China. The active prevention and treatment of the associated complications, including sarcopenia, in diabetes patients and the improvement of their quality of life will be the focus of future research in China [16].

Timely recognition of sarcopenia is important because it is amenable to intervention, especially when diagnosed early. Screening tools may facilitate its diagnosis for sarcopenia and, thereby, management, especially for high-risk population. For these reasons, a routine clinical screening tool for sarcopenia in type 2 diabetes patients that is easy to operate is required. Unfortunately, only a few studies have examined the association of sarcopenia in type 2 diabetes in China. Therefore, we designed a study to develop a simple screening tool for sarcopenia and to examine its ability to estimate the probability of sarcopenia based on a multicenter, cross-sectional study, which had been designed to estimate the prevalence of sarcopenia in type 2 diabetes adults and had been conducted in several hospitals in Beijing, China. In this study, a total of 1125 participants were randomly divided into two groups: an exploratory population and a validation population. A sarcopenia risk score was developed based on the multivariable logistic regression analysis of the exploratory population to identify earlier the probability of sarcopenia in type 2 diabetes patients. This sarcopenia risk score was then validated in the validation population, aiming to evaluate whether, as a new alternative screening tool, it was suitable to screen for sarcopenia in Chinese adults with type 2 diabetes.

## Research design and methods

### Study sample

We followed the methods of He et al. [17]. Data for the development of the new sarcopenia risk score were collected from a multicenter, cross-sectional survey study designed to estimate the prevalence of sarcopenia in type 2 diabetes adults recruited from the endocrinology department of nine different hospitals in Beijing, China, from January 2016 to March 2018. The nine research centers consisted of five urban hospitals and four suburban hospitals selected by a random sampling method. The inclusion criteria were patients aged 50 years or older with a previous diagnosis of type 2 diabetes. Known diabetes was defined as the self-report of a diabetes diagnosis or based on treatment information or the documentation of the plasma glucose level in medical records. According to the World Health Organization definition, previously diagnosed type 2 diabetes was defined as having either fasting plasma glucose (FPG) ≥ 7.0 mmol/L or 2-h postprandial glucose (2hPG) ≥ 11.1 mmol/L. The exclusion criteria included the following: (1) patients with serious systemic diseases, including severe hepatic insufficiency, moderate to severe renal insufficiency, or cardiac insufficiency; (2) patients with tuberculosis; (3) patients with severe depression, schizophrenia and other mental illness; (4) patients with cognitive disability and those who were unable to record dietary information in the diary or to cooperate with the examination; (5) patients with in vivo metal stent implantations or pacemakers, which would affect the accuracy of the body composition analysis; (6) patients who had undergone weight loss surgery; and (7) patients with type 1 diabetes mellitus, secondary diabetes mellitus or gestational diabetes mellitus. The study was approved by the Beijing Hospital Ethics Committee (Approval No. 2015BJYYEC-052-02), and informed consent was obtained from all participants. A total of 1125 participants aged 50 years or older met the aforementioned criteria and were included in the current data analyses. All populations were randomly divided into two equal groups: an exploratory population and a validation population. A multivariable logistic regression analysis was conducted in the exploratory population to establish the new sarcopenia risk score and applied the internal validation. An external reliability test was conducted in the validation population.

### Questionnaire survey

A standardized questionnaire was designed to collect patient information, including demographic data such as birth date, sex, habits and customs, alcohol drinking status (current drinker or not), smoking status (current smoker or not), the time since the onset of diabetes mellitus, and medication for diabetes, including oral hypoglycemic drugs or insulin. In addition, information on some chronic complications of diabetes, including cardiovascular disease, hypertension, diabetic peripheral vascular disease, diabetic peripheral neuropathy, and diabetic nephropathy, was recorded from self-reports or medical records. There was a team consisting of endocrinologists, nutritionists, educational nurses and check-up physicians involved in the study at each research center. The endocrinologist was responsible for data collection, and the nutritionist and educational nurses were in charge of dietary records and analysis. The check-up physician was responsible for anthropometric and body composition measurements. A standardized study protocol manual was distributed to every researcher, and all investigators had to receive unified training before the study to reduce bias among researchers.

### Anthropometric measurements and body composition

Body weight and height were measured using a digital floor scale and a wall-mounted stadiometer to the nearest 0.1 cm and 0.1 kg, respectively, with the patient in light clothes and without shoes. Body mass index (BMI) was calculated as the weight in kilograms divided by the square of the height in meters (kg/m^2^). Body composition was determined by a bioimpedance analyzer using Inbody 720 (Biospace, Korea). Body weight and body composition assessment was measured at early morning fasting for at least 10 h. We recorded trunk muscle mass, appendicular and whole-body skeletal muscle mass, fat mass and the percentage of body fat. The appendicular skeletal muscle mass index (ASMI) was calculated by dividing the appendicular skeletal muscle mass by the height squared (kg/m^2^). Handgrip strength was measured two times in each hand by an electronic hand dynamometer (EH101, Zhejiang Province, China), and the maximum value was recorded.

### The cutoff point for the diagnosis of sarcopenia

The criteria for the diagnosis of sarcopenia required the coexistence of low muscle mass and low muscle strength according to the recommendation of the Asian Working Group for Sarcopenia (AWGS) 2014 [8]. Low muscle mass was determined as an ASMI below the lower quintile of the homogeneous, same sex, healthy young reference group. The cutoff points of ASMI were calculated in healthy young staff members of Beijing Hospital in China who received a routine health check-up and body composition examination by a bioimpedance analyzer. This cohort included 402 volunteers who were doctors and nurses (102 males and 300 females), aged between 18 and 35 years old, with a BMI between 18.5 kg/m^2^ and 24.0 kg/m^2^. Their ASMIs were calculated, and the lower quintile of ASMI was 7.18 kg/m^2^ and 5.73 kg/m^2^ in men and women, respectively. The subjects with an ASMI less than 7.18 kg/m^2^ in men or 5.73 kg/m^2^ in women were considered to have low muscle mass. Low muscle strength was defined as a grip strength below the lower quintile of the same sex subjects in this study, and the lower quintile cutoff value of muscle strength was 29.5 kg for males and 21.2 kg for females.

### Laboratory measurements

The study participants were instructed to maintain an overnight fast of at least 10 h before blood samples were collected. Total cholesterol (TC), triglycerides (TG), high-density lipoprotein cholesterol (HDL-c) and low-density lipoprotein cholesterol (LDL-c) were measured by an automatic biochemical analyzer (Beckman Coulter AU5400, USA). Blood glucose levels were determined by the glucose oxidase method. Glycated hemoglobin A1c (HbA1c) was measured by high-performance liquid chromatography (HPLC) (Premier HB9210, Trinity Biotech, Kansas, USA).

### Dietary records and analysis

All participants had to keep diet diary for consecutive three days, including two working days and one weekend day, in accordance with the guidance of the nutritionist and educational nurses. The weight of each type of food that was eaten in the three days, including staple foods, vegetables, meats and snacks, was recorded. We calculated the total energy intake, carbohydrate, protein, and fat intake per day and the proportion of calorific energy supplied by protein, carbohydrate and fat by nourishment analysis software (V4.0.3, Zhending Health Technology Co., Shanghai, China). The total energy was adjusted by body weight, which was calculated by the total energy daily (kcal) divided by the ideal body weight (kilograms). The ideal body weight was calculated by the height (centimeter) minus 105 [18].

### Statistical analysis

Statistical analysis was performed using SPSS for Windows, version 18.0 (SPSS, Chicago, IL). Normal distribution was tested for all parameters. Normally distributed continuous data are presented as the means ± standard deviation, and categorical variables are presented as numbers and percentages (%). An independent-sample t test was used to compare the means of two groups, and a Chi-squared test was used to compare the percentages. Skewed distribution data were presented as the median (25–75th percentile) and tested by nonparametric test. A multivariable logistic regression model using the backward stepwise likelihood ratio method to estimate the probability of sarcopenia was fitted with candidate variables including age, sex, BMI, diabetes duration, glycosylated hemoglobin, treatment regimen, nutrient intake per day and chronic complications of diabetes mellitus, current drinking and current smoking. A new sarcopenia risk score created based on the rounded values of the shrunken regression coefficients of the significant variables was presented to facilitate clinical application. The Hosmer–Lemeshow goodness-of-fit test was used to investigate how close the prevalence predicted to the observed prevalence. The difference was considered nonsignificant at *P* > 0.05. The validation of the sarcopenia risk score developed from the exploratory population was conducted in the validation population. The ability of the model to correctly rank order participants by sarcopenia probability (discrimination ability) was assessed by the area under the receiver operator characteristic (ROC) curve, and C statistics were calculated to compare the AUCs. The ROC curve was obtained by plotting sensitivity against 1-specificity at each cutoff value stratified by gender. The optimal cutoff point was identified using the Youden index, which was at the maximum sum of the sensitivity and specificity-1. The positive and negative predictive values were calculated at the cutoff point.

## Results

### Baseline characteristics and the prevalence of sarcopenia among participants

Table 1 shows the baseline characteristics of participants in the exploratory population and validation population with and without sarcopenia.

**Table 1:** Baseline characteristics of participants in the exploratory population and validation population with or without sarcopenia

	Exploratory population			Validation population		
	No sarcopenia	With sarcopenia	*P*	No sarcopenia	With sarcopenia	*P*
Men						
N	254	44		257	31	
Age (years)	61.4 ± 8.1	67.4 ± 10.2	0.001	61.6 ± 8.1	70.4 ± 10.2	< 0.001
Duration of diabetes (years)	10.2(5.3, 14.9)	10.0(5.6,18.2)	0.247	10.5(5.3,16.9)	11.2(5.3,19.8)	0.999
BMI (kg/m^2^)	26.0 ± 2.9	23.3 ± 2.8	< 0.001	26.3 ± 3.6	23.0 ± 2.7	< 0.001
Current smoking, *n* (%)	98(38.6%)	15(34.1%)	0.571	87(34%)	9(29%)	0.577
Current drinking, *n* (%)	134(52.8%)	18(40.9%)	0.147	118(45.9%)	12(38.7%)	0.568
Diabetic nephropathy, *n* (%)	98(38.6%)	17(14.8%)	0.995	95(37%)	17(54.8%)	0.083
Diabetic peripheral vascular disease, *n* (%)	146(57.5%)	28(63.6%)	0.549	153(59.5%)	23(74.2%)	1.114
Diabetic peripheral neuropathy, *n* (%)	108(42.5%)	25(56.8%)	0.078	109(42.4%)	19(61.3%)	0.071
Hypertension, *n* (%)	164(64.6%)	27(61.4%)	0.683	152(59.1%)	18(58.1%)	0.908
Cerebrovascular disease, *n* (%)	34(13.4%)	4(9.1%)	0.43	41(16%)	10(32.3)	0.046
Coronary heart disease, *n* (%)	50(19.7%)	10(22.7%)	0.642	39(15.2%)	8(25.8%)	0.13
Treatment regimen						
Insulin, *n* (%)	104(40.9%)	15(34.1%)	0.391	122(47.5%)	14(45.2%)	0.808
Oral hypoglycemic agents, *n* (%)	187(73.6%)	35(79.5%)	0.405	180(70%)	20(64.5%)	0.671
FPG (mmol/L)	8.5 ± 2.4	8.2 ± 2.7	0.449	8.4 ± 2.5	8.4 ± 2.6	0.942
HbA1c (%)	7.9 ± 1.7	8.2 ± 1.4	0.307	7.9 ± 1.6	8.9 ± 2	0.002
Triglycerides (mmol/L)	1.5(1.0,2.4)	1.1(0.7,1.7)	< 0.001	1.4(1.0,2.1)	1.0(0.8,1.5)	0.002
Total cholesterol (mmol/L)	4.3 ± 1.0	4.3 ± 1.3	0.839	4.3 ± 1.1	4.2 ± 0.8	0.455
HDL-C (mmol/L)	1.1 ± 0.3	1.3 ± 0.4	0.005	1.1 ± 0.3	1.2 ± 0.4	0.289
LDL-C (mmol/L)	2.6 ± 0.8	2.5 ± 0.9	0.676	2.7 ± 0.9	2.6 ± 0.9	0.738
Total energy intake (kcal/kg/d)	29.4 ± 7.6	30.8 ± 8.6	0.291	29.9 ± 7.8	29.4 ± 9	0.779
Proportion of calories supplied by protein (%)	15.9 ± 3.2	14.8 ± 2.5	0.026	16.2 ± 4.5	15.1 ± 2.9	0.205
Proportion of calories supplied by carbohydrate (%)	53.5 ± 8.0	53.5 ± 6.8	0.976	53.9 ± 8.5	53.4 ± 6.4	0.747
Proportion of calories supplied by fat (%)	30.6 ± 7.3	31.8 ± 6.4	0.338	30.1 ± 7.6	31.6 ± 6.2	0.301
Body fat percentage (%)	26.6 ± 5.6	28.3 ± 7.9	0.185	26.5 ± 6.1	28.5 ± 7.5	0.108
ASMI (kg/m^2^)	8.0 ± 0.6	6.7 ± 0.5	< 0.001	8.1 ± 0.7	6.6 ± 0.6	< 0.001
Grip strength (kg)	33.7 ± 6.5	27 ± 3.7	< 0.001	34.2 ± 7.5	25.8 ± 5.1	< 0.001
Women						
N	247	11		271	10	
Age (years)	62.6 ± 7.9	65.7 ± 9.8	0.206	62.5 ± 7.4	68.1 ± 8.3	0.021
Duration of diabetes (years)	10.3(5.4,15.4)	12.1(5.8,19.7)	0.584	10.3(5.2,15.6)	12.2(8.7,16.9)	0.359
BMI (kg/m^2^)	25.8 ± 3.1	22.6 ± 4.2	0.001	26.3 ± 3.3	22.1 ± 2.0	< 0.001
Current smoking, *n* (%)	8(3.2%)	1(9.1%)	0.329	8(3.0%)	0(0%)	0.443
Current drinking, *n* (%)	19(7.7%)	0(0%)	0.339	16(5.9%)	1(10%)	0.623
Diabetic nephropathy, *n* (%)	82(33.2%)	2(18.2%)	0.477	82(30.3%)	1(10.0%)	0.305
Diabetic peripheral vascular disease, *n* (%)	127(51.4%)	6(54.5%)	0.839	144(53.1%)	3(30.0%)	0.264
Diabetic peripheral neuropathy, *n* (%)	100(40.5%)	5(45.5%)	0.743	114(42.1%)	4(40.0%)	0.897
Hypertension, *n* (%)	126(51%)	7(63.6%)	0.412	163(60.1%)	7(70.0%)	0.531
Cerebrovascular disease, *n* (%)	27(10.9%)	1(9.1%)	0.848	33(12.2%)	3(30.0%)	0.098
Coronary heart disease, *n* (%)	46(18.6%)	3(27.3%)	0.474	40(14.8%)	2(20%)	0.648
Treatment regimen						
Insulin, *n* (%)	94(38.1%)	3(27.3%)	0.47	105(38.7%)	2(20%)	0.231
Oral hypoglycemic agents *n* (%)	192(77.7%)	7(63.6%)	0.276	206(76%)	9(90%)	0.519
FPG (mmol/L)	8.4 ± 2.7	9.1 ± 3.8	0.392	8.6 ± 2.8	7.1 ± 1.3	0.081
HbA1c (%)	8.1 ± 1.8	8.5 ± 1.8	0.047	8.3 ± 2	8.7 ± 1.2	0.047
Triglycerides (mmol/L)	1.6(1.0,2.4)	1.3(0.8,1.9)	0.362	1.5(1.1,2.1)	1.5(0.8,2.5)	0.612
Total cholesterol (mmol/L)	4.8 ± 1.3	5.5 ± 1.6	0.079	4.7 ± 1.2	4.7 ± 0.8	0.963
HDL-C (mmol/L)	1.2 ± 0.3	1.5 ± 0.4	0.001	1.2 ± 0.3	1.3 ± 0.4	0.578
LDL-C (mmol/L)	2.9 ± 1.0	3.1 ± 1.3	0.393	2.9 ± 0.9	2.8 ± 0.7	0.791
Total energy intake (kcal/kg/d)	29.6 ± 6.9	29.7 ± 5.5	0.971	30.0 ± 7.1	30.7 ± 7.9	0.753
Proportion of calories supplied by protein (%)	15.1 ± 3.6	14.0 ± 3.1	0.35	15.3 ± 4.5	16.0 ± 1.6	0.647
Proportion of calories supplied by carbohydrate (%)	54.4 ± 7.4	51.5 ± 9.5	0.218	53.7 ± 7.2	54.2 ± 7.8	0.833
Proportion of calories supplied by fat (%)	30.8 ± 6.1	34.1 ± 7.9	0.089	31.4 ± 6.5	29.9 ± 7	0.476
Body fat percentage (%)	34.7 ± 5.6	33.6 ± 10.1	0.534	36.1 ± 5.8	34.5 ± 5.3	0.384
ASMI (kg/m^2^)	6.7 ± 0.8	5.3 ± 0.3	< 0.001	6.7 ± 0.7	5.4 ± 0.2	< 0.001
Grip strength (kg)	26.2 ± 4.4	16.2 ± 2.9	< 0.001	25.8 ± 4.4	17.1 ± 2.8	< 0.001

Data are mean ± SD, *n* (%), or median (25–75th percentile) unless otherwise indicated

A total of 556 type 2 diabetes patients, including 298 men and 258 women, were analyzed in the exploratory population. The average age was 62.3 ± 8.7 years in men and 62.7 ± 7.9 years in women. The BMI was 25.6 ± 3.1 kg/m^2^ and 25.7 ± 3.2 kg/m^2^, the median duration of diabetes was 10.1 years and 10.4 years, the fasting plasma glucose was 8.4 ± 2.4 mmol/l and 8.4 ± 2.8 mmol/l, and HbA1c was 7.9 ± 1.6% and 8.1 ± 1.8% in men and women, respectively. The prevalence of sarcopenia was 14.8% in men and 4.3% in women. In the validation population, the participants did not differ much from the exploratory population in terms of age, the duration of diabetes, chronic complications of diabetes mellitus, fasting plasma glucose, or glycosylated hemoglobin. The prevalence of sarcopenia was higher in the exploratory population than in the validation population (14.8% vs 10.8% in men, 4.3% vs 3.6% in women), but the difference was not significant.

When compared to no-sarcopenia participants, the with-sarcopenia participants were older and had lower BMI, higher glycosylated hemoglobin (HbA1c), lower appendicular skeletal muscle mass index (ASMI) and muscle strength in both genders. Furthermore, dietary intake analysis demonstrated that participants with sarcopenia had higher total energy intake per day and lower proportion of calories supplied by protein.

### The multivariable model from the logistic regression analysis in the exploratory population

The Box–Tidwell method test for linearity suggested that the continuous independent variables were linearly associated with the logit of sarcopenia probability. After the variable selection procedure, the final logistic model to estimate the probability of sarcopenia included five variables (Table 2) and had statistical significance (*χ*^2^ = 79.074, *P* = 0.000). In the multivariable logistic regression analysis, increased age, male, low BMI, total energy intake per day, and the proportion of calories supplied by protein were significantly associated with the prevalence of sarcopenia in type 2 diabetes. The AUCs of the predicted prevalence of sarcopenia in type 2 diabetes of the multivariable model were 0.818 (95% CI 0.756–0.880, *P* = 0.000). The Hosmer–Lemeshow test showed that the multivariable model matched well with the observed prevalence (*χ*^2^ = 5.503, *P* = 0.703).

**Table 2:** Odds ratios (95% CI) and *β*-coefficients for the prevalence of sarcopenia among diabetes patients in the exploratory population estimated using logistic regression analysis

	*β*-Coefficient	S.E	Wald	OR (95% CI)
Gender	1.563	0.369	17.931	4.775 (2.316, 9.847)
Age (years)	0.784	0.175	20.205	2.191 (1.556, 3.085)
BMI (kg/m^2^)	1.278	0.256	24.887	3.588 (2.172, 5.927)
Total energy intake (kcal/kg/d)	0.042	0.02	4.506	1.043 (1.003, 1.085)
Proportion of calories supplied by protein (%)	− 0.126	0.061	4.265	0.881 (0.782, 0.994)

### The new, alternative risk score for the probability of sarcopenia in Chinese diabetes patients based on the multivariable model

Table 3 shows the new, alternative sarcopenia risk score developed based on the rounded values of the shrunken regression coefficients of the five variables. The total scores ranged from − 2 to 19. The optimal cutoff points for the predicted prevalence of sarcopenia were 12 points, 12 points and 10 points in all individuals, men and women, respectively. The maximal Youden index was 0.519, 0.502 and 0.363, in all individuals, men and women, respectively. The sensitivity and specificity were 70.9% and 81.0% in all individuals, 84.1% and 66.1% in men, and 54.5% and 81.8% in women, respectively. The positive predictive value and negative predictive value were 33.0% and 94.4% in all individuals, 34.1% and 92.6% in men and 15.4% and 97.0% in women, respectively. A total of 88 (15.8%) participants in the exploratory population had a risk score ≥ 12, and 29 (33.0%) had sarcopenia. The AUCs of the ROC curve based on the exploratory population were 0.806 (95% CI 0.741–0.872), 0.797 (95% CI 0.725–0.869), and 0.700 (95% CI 0.532–0.868) in all individuals, men and women, respectively.

**Table 3:** The sarcopenia risk score for the probability of sarcopenia among diabetes patients based on the logistic regression analysis

Characteristic	Scores
Gender	
Women	0
Men	4
Age (years)	
50–59	0
60–69	2
70–79	4
≥ 80	6
BMI (kg/m^2^)	
≥ 28	0
24–27.9	4
< 23.9	7
Total energy intake (kcal/kg/d)	
12.4–22.4	0
22.5–32.4	1
32.5–42.4	2
42.5–52.4	3
≥ 52.5	4
Proportion of calories supplied by protein (%)	
< 18	0
19–28	− 2
29–38	− 4
39–48	− 6
49–61.2	− 8

**Fig. 1:**
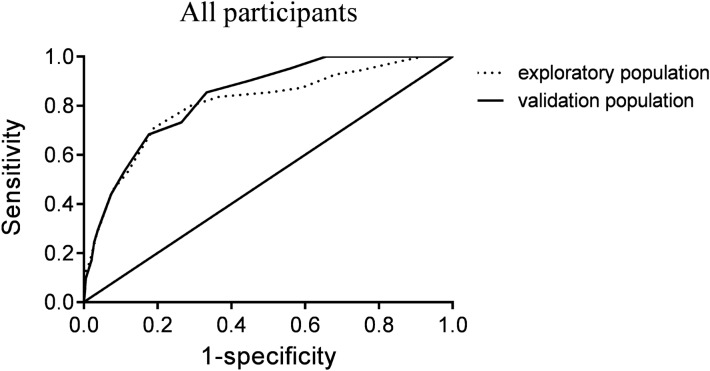
Shows the ROC in all participants. The receiver operator characteristic (ROC) curve of the sarcopenia risk score for the prediction of the probability of sarcopenia in all participants, men and women based on the exploratory and validation populations

**Fig. 2:**
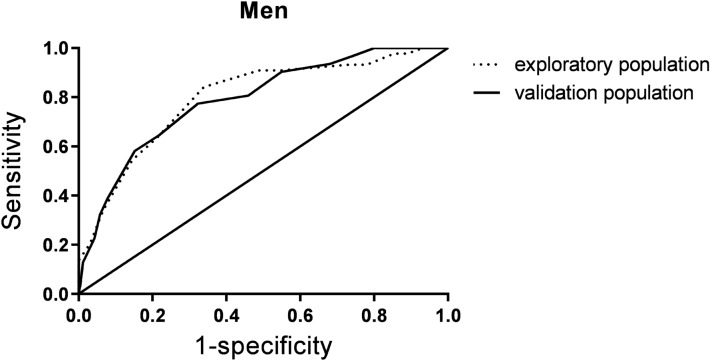
Shows the ROC in male participants. The receiver operator characteristic (ROC) curve of the sarcopenia risk score for the prediction of the probability of sarcopenia in all participants, men and women based on the exploratory and validation populations

**Fig. 3:**
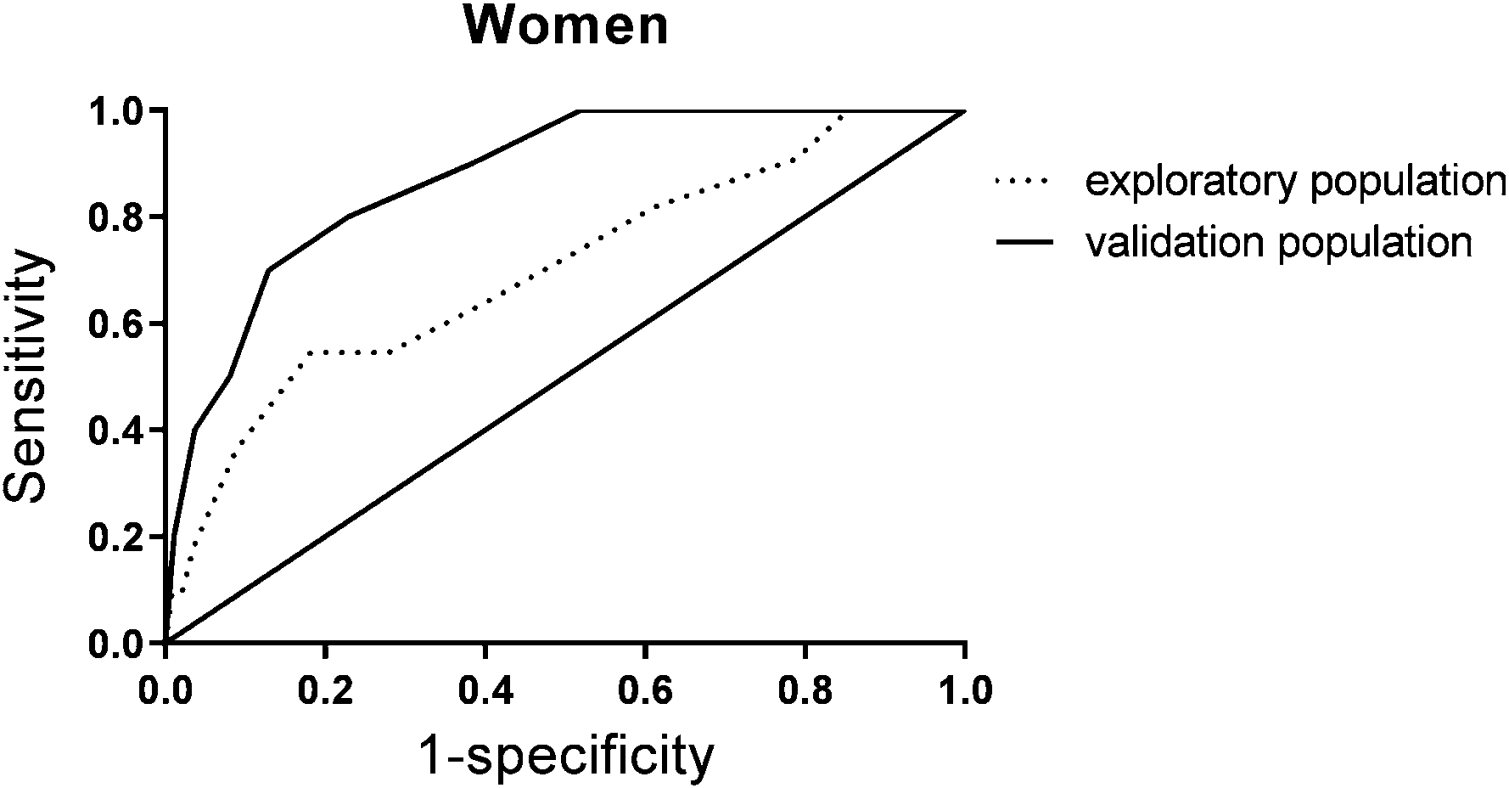
Shows the ROC in female participants. The receiver operator characteristic (ROC) curve of the sarcopenia risk score for the prediction of the probability of sarcopenia in all participants, men and women based on the exploratory and validation populations

The AUCs of the risk score for the prediction of the incidence of sarcopenia in type 2 diabetes patients based on the validation population were 0.836 (95% CI 0.781–0.892), 0.789 (95% CI 0.707–0.871), and 0.873 (95% CI 0.782–0.965) in all participants (Fig. 1), men (Fig. 2), and women (Fig. 3), respectively. A total of 81 (14.2%) individuals in the validation population had a risk score ≥ 12 points, and 22 (27.2%) had sarcopenia. At a cutoff point ≥ 12, the sensitivity and specificity in the validation population were 53.7% and 88.8% in all individuals, and the positive predictive value and negative predictive value were 27.2% and 96.1%, respectively. The Hosmer–Lemeshow goodness-of-fit test showed a good calibration with the risk score in external validation (*χ*^2^ = 4.459, P = 0.813). At a cutoff point ≥ 12, the sensitivity and specificity were 64.5% and 78.2% in men, and the positive and negative predictive values were 26.3% and 94.8%, respectively. At a cutoff point ≥ 10, the sensitivity and specificity were 50.0% and 91.9% in women, and the positive and negative predictive values were 18.5% and 98.0%, respectively.

The diagnostic accuracy indicators of the sarcopenia risk score in the exploratory and validation populations are shown in Table 4.

**Table 4:** Diagnostic accuracy indicators of the sarcopenia risk score in the exploratory and validation populations

	AUC	95% CI	Youden index	Cutoff point	Sensitivity (%)	Specificity (%)	Positive predictive value (%)	Negative predictive value (%)
Exploratory population								
All	0.806	0.741–0.872	0.519	12	70.9	81.0	33.0	94.4
Men	0.797	0.725–0.869	0.502	12	84.1	66.1	34.1	92.6
Women	0.700	0.532–0.868	0.363	10	54.5	81.8	15.4	97.0
Validation population								
All	0.836	0.781–0.892			53.7	88.8	27.2	96.1
Men	0.789	0.707–0.871			64.5	78.2	26.3	94.8
Women	0.873	0.782–0.965			50.0	91.9	18.5	98.0

## Discussion

Type 2 diabetes is associated with an increased risk of sarcopenia, low muscle mass and low muscle strength [19]. To estimate the probability of sarcopenia in functionally independent Chinese type 2 diabetes adults, we created a multivariate model based on the seven selected variables. Logistic regression analysis showed that male, low BMI, aging, low protein intake and excessive daily energy intake were risk factors that were independently associated with sarcopenia in type 2 diabetes. The AUC of the multivariable model was 0.818 (95% CI 0.756–0.880), which illustrated an excellent discrimination ability, and the Hosmer–Lemeshow test showed good fitness of the model. These results implied that type 2 diabetes patients in China would lose greater skeletal muscle mass and strength with aging, hyperglycemia and excessive total energy intake and less protein intake, which were in agreement with several previous studies [12, 20]. The risk of sarcopenia in type 2 diabetes would decline with good control of glucose levels, a restriction of total energy intake, an increase in protein intake and the maintenance of a moderate BMI. The early identification of sarcopenia in type 2 diabetes would help clinicians to provide treatment as early as possible, which would be beneficial to improve the prognosis and quality of life of type 2 diabetes patients [21].

To facilitate clinical application, a new sarcopenia risk score for the detection of sarcopenia in type 2 diabetes patients comprising age, gender, BMI, total energy intake per day and the proportion of calories supplied by protein was developed based on our data. The performance of the new sarcopenia risk score with high diagnostic accuracy was adequate for the detection and prediction of sarcopenia in Chinese type 2 diabetes patients aged 50 years and older. In our findings, the AUCs of the ROC curve representing the concordance statistics were 0.806 (95% CI 0.741–0.872) and 0.836 (95% CI 0.781–0.892) in internal and external validation, respectively, showing that the new sarcopenia risk score had good discrimination ability to identify high-risk patients with sarcopenia among those with type 2 diabetes. The Hosmer–Lemeshow goodness-of-fit test in external validation showed that the new sarcopenia risk score had high accuracy to distinguish sarcopenia patients.

In this study, the age of the participants included was 50 years or older. It was reported that loss of muscle mass and strength become pronounced around the age of 50, progressed faster after the age of 60. Therefore, AWGS recommended screening for sarcopenia among community-dwelling older people as well as older people with certain clinical conditions such as diabetes mellitus, which was associated with increased risk of developing sarcopenia. For this reason, late middle age is probably a good time to identify sarcopenia so that it can be stabilized or reversed in time to prevent adverse outcomes.

This is an initial exploratory research aimed to establish a simple screening tool easy to operate and early to identify sarcopenia from type 2 diabetes patients. According to the current consensuses, diagnostic criteria from the EWGSOP [3] or AWGS [8] are still the operational criteria for the diagnosis of sarcopenia; however, there are some shortcomings in the diagnosis process. The results of the diagnostic criteria were device-dependent and time-consuming and could differ based on the ethnic diversity of the cutoff points and the possible bias induced by body size and shape variations. Many medical institutions in China had no body composition instruments, and the X-ray exposure of dual-energy X-ray absorptiometry and high cost of computed tomography and magnetic resonance imaging restricted their widespread use in clinical practice. For this reason, the new, alternative sarcopenia risk score in our study was suitable for the detection of sarcopenia in type 2 diabetes patients when body composition could not be examined, had the advantage of being free of X-ray exposure and was a more practical and inexpensive choice than computed tomography, magnetic resonance imaging, or dual-energy X-ray absorptiometry. It was not difficult to acquire the parameters of the new, alternative sarcopenia risk score. BMI could be calculated by measuring height and weight, nutrient intake could be calculated by nourishment analysis software by reviewing the dietary diary. Furthermore, different from the SARC-F [5] and the Mini Sarcopenia Risk Assessment (MSRA) [6], which were developed and validated based on community-dwelling older adults, the new, alternative sarcopenia risk score in our study specialized in the population with diabetes, including nursing home residents or hospitalized older adults, who were at high risk of sarcopenia. Type 2 diabetes patients with sarcopenia were good candidates for intervention to prevent further physical disability given their potential for regaining muscle mass and the restoration of muscle function. In conclusion, the new, alternative sarcopenia risk score in our study was an effective and convenient health promotion tool instead of a diagnostic test and could become an alternative tool for endocrinologists to screen for sarcopenia in patients with type 2 diabetes.

The present study had several limitations that must be addressed. Firstly, in the current study, the sample size of sarcopenia patients was small because of a low prevalence of sarcopenia, which would have a negative impact on the statistical power. An enlarged sample will be needed in future study and further validation of the risk score in other parts of China is needed given the large diversity of the Chinese population. Secondly, the current analysis was carried out on a population of Chinese type 2 diabetes adults. Therefore, our findings might not generalize to populations that have no diabetes mellitus, and those of other races/ethnicities or those in other countries. Future studies will be designed to test their performance and usability in different populations and settings. Thirdly, we had not evaluate physical performance in this study, which would underestimate the detection rate of sarcopenia. While EWGSOP advised to confirm sarcopenia by detection of low muscle quantity and quality, to determine severity of sarcopenia by evaluate physical performance. Sarcopenia with low physical performance is considered severe. In the current study, we aimed to create a screening tool for sarcopenia in Chinese patients with type 2 diabetes in clinical practice. The coexistence of low muscle mass and low muscle strength could be made a diagnosis of sarcopenia, so it would not impair the detection rate of sarcopenia. Lastly, dietary diary was self-reported, there may be a potential for interindividual errors. Dietary diary is a research challenge. In order to diminish errors, all participants had to receive training when included in the study on how to keep a dietary diary correctly and how to estimate the weight of food accurately. All participants had to record dietary diary detailed for consecutive three days to take an average to reduce daily errors. Since most of middle aged and elderly people usually have a stable dietary habits and customs, dietary assessment can speculate the patient's nutritional intake status.

## Conclusion

In summary, this study developed a new, alternative sarcopenia risk score to identify sarcopenia among patients with type 2 diabetes and demonstrated internal consistency and high accuracy. The new, alternative sarcopenia risk score can be used as an effective screening tool and has been validated to identify functionally independent type 2 diabetes adults at high risk of sarcopenia who are good candidates for intervention to prevent further physical disability in clinical practice in China.

## Data Availability

The research data used to support the findings of this study are available from the corresponding author upon request. The corresponding author email: 13811620484@163.com.
